# Draft Genome Sequence of the Cadmium-Resistant Strain JJU2, Belonging to the Family Hapalosiphonaceae of the Cyanobacteria

**DOI:** 10.1128/MRA.00876-18

**Published:** 2018-08-30

**Authors:** Angelo Joshua Victoria, Ernelea Cao, Nico Salmaso, Nicola Segata, Claudio Donati

**Affiliations:** a Institute of Biology, University of the Philippines Diliman, Quezon City, Philippines; b Research and Innovation Centre, Fondazione Edmund Mach, San Michele all’Adige, Italy; c Centre for Integrative Biology, University of Trento, Trento, Italy; Indiana University Bloomington

## Abstract

Here, we report the genome of strain JJU2, a cyanobacterium of the family *Hapalosiphonaceae* known to be resistant to high cadmium levels, assembled from a nonaxenic, unialgal culture from Marinduque, Philippines. The draft genome is 7.1 Mb long with a GC content of 40.05% and contains 5,625 protein-coding genes.

## ANNOUNCEMENT

The cyanobacterium strain JJU2 is a member of the family *Hapalosiphonaceae* (phylum *Cyanobacteria*) characterized by heterocyte formation and true branching (1). Evidence of cadmium tolerance mechanisms in strain JJU2 were previously reported (2). However, little is known about the molecular genetics of members of the *Hapalosiphonaceae* family, and to date, there are only two genomes sequenced for the genus in public databases, Hapalosiphon welwitschii UC-IC-52-3 (3) and Hapalosiphon sp. strain MRB220 (4). Differences in secondary metabolite gene clusters were reported by these studies, but the genetic underpinnings of adaptations to metal tolerance are still lacking. To expand the genomic representation of the family *Hapalosiphonaceae*, we report here the genome of strain JJU2, isolated from the heavy-metal-contaminated Mogpog River in Marinduque, Philippines.

Strain JJU2 was isolated from a freely floating colony grown from the isolation of a single filament. The unialgal, nonaxenic culture was maintained in BG-11 medium with a 12-hour light-dark cycle at the Plant Genetics and Cyanobacterial Biotechnology Laboratory of the University of the Philippines, Diliman Institute of Biology. The sample was repeatedly washed with sterile BG-11 medium (5) and filtered through a 0.45-µm membrane (Merck Millipore, USA) prior to extraction using the ZR bacterial/fungal DNA miniprep kit (Zymo, USA). Library preparation and 150-bp paired-end sequencing were performed at the Philippine Genome Center DNA Sequencing Core Facility on the MiSeq platform (Illumina, USA).

Raw sequencing reads were quality trimmed and filtered using FASTX-Toolkit 0.0.13 (6). Contaminant reads were identified by performing k-mer analysis (7) using K-mer Analysis Toolkit 2.3.2 (8) and khmer 2.1.2 (9) (k = 45) and removed using Bowtie 2 2.3.4.1 (10). The 13,891,516 filtered reads were assembled using SPAdes 3.11.0-1 (11) with default parameters and the error-correction pipeline enabled, generating an initial assembly of 1,084 contigs. The genome was predicted by CheckM (12) to be almost complete (96.84%) with virtually no contamination or strain heterogeneity. Scaffolding was performed using MeDuSa (13) with default parameters.

The final assembled 7,145,111-bp-long genome comprised 209 scaffolds with an *N*_50_ value of 89,291 bp, a GC content of 40.05%, and a coverage of 111×. Annotation and biosynthetic gene cluster prediction was performed with the NCBI Prokaryotic Genome Annotation Pipeline (14) and antiSMASH 4.1.0 (15) using default parameters, identifying 5,625 coding sequences and 41 tRNAs. Genes for cadmium tolerance, such as *czcA*, *czcB*, and *cadA*, were annotated, as were 30 other heavy metal tolerance genes, including the metallothionein *smtA* and its known transcriptional repressor, *smtB*. Prediction of biosynthetic gene clusters revealed that this cyanobacterium has gene clusters for cyanotoxin production with 30% and 27% similarities to microcystin and nostophycin, respectively. It also encodes a polyketide synthase gene cluster with 90% and 71% identities to the ambiguine and welwitindolinone natural-product gene clusters, respectively, highlighting the potential of this species for sensing and adaptation to metal stress and production of toxic metabolites.

### Data availability.

This whole-genome shotgun project has been deposited at DDBJ/ENA/GenBank under the accession number QLKN00000000. The version described in this paper is version QLKN01000000.
